# Fungal communities associated with *Heterodera glycines* and their potential in biological control: a current update

**DOI:** 10.21307/jofnem-2020-022

**Published:** 2020-03-17

**Authors:** Deepak Haarith, Kathryn E. Bushley, Senyu Chen

**Affiliations:** 1 Department of Plant Pathology, University of Minnesota, St. Paul, MN 55108; 2 Department of Plant and Microbial Biology, University of Minnesota, St. Paul, MN 55108

**Keywords:** Soybean cyst nematode, SCN, Biocontrol, Biological control, Fungi, Fungal metabolites, Mycobiome, *Heterodera* spp., *Heterodera glycines*, Review

## Abstract

The soybean cyst nematode (SCN) is the most important pest on soybean, a major crop worldwide. The SCN is considered both parasitic and pathogenic as it derives nutrition from the host and manipulates host physiology to do so. Currently, there are no commercially available chemicals that are specific, environmentally safe and cost effective to control SCN levels. Crop rotation, use of host resistance and other cultural practices remain the main management strategies. The need for bioprospecting other methods of controlling SCN is paramount, and fungi show promise in that respect. Several studies have evaluated fungi and fungal products as biocontrol options against plant-parasitic nematodes. This review discusses fungal genera isolated from the SCN with potential for use as biocontrol agents and the effects of their secondary metabolites on various stages of SCN development. The review also summarizes efforts to control SCN using soil amendments that could potentially impact fungal communities in the soil.

The soybean cyst nematode (*Heterodera glycines*, SCN) was discovered as a significant pest of soybean in 1954 in North Carolina in the USA, and subsequently spread across a few counties in Arkansas, Missouri, Kentucky and Tennessee, as well as few other pockets in North Carolina (Winstead and Skotland, 1955). In 2014, bolstered by the increase in planting of soybean, the SCN has become a significant plant health issue all over the agrarian eastern continental United States as well as Hawaii and Puerto Rico, and southern Canada (Tylka and Marett, 2014). In the USA, SCN accounts for up to 30% of all soybean yield lost to disease, amounting to a little over 2.7 m metric tons a year (Allen et al., 2017). It is a major problem of this important protein and oilseed crop of the USA, the largest producer of soybean with an annual output of 117.3 m metric tons in 2016 (White and Honig, 2017).

The lifecycle of SCN includes several life stages that can be targeted by natural antagonists. However, the survival structures or cysts, which consist of the female bodies containing hundreds of eggs protected by a melanized layer of cuticle, are an important target stage for biocontrol. The eggs in cysts can survive up to 10 years or longer in soil and can produce viable and infective second-stage juvenile nematodes (J2) when conditions are favorable. Thus, reducing the egg numbers in cysts in soil will ensure significant reduction in nematode inoculum.

The idea of using biological agents to control nematodes is as old as nematology itself, as Nathan Cobb, the father of nematology, first suggested using predatory nematodes to control plant-parasitic nematodes (Cobb, 1917). Biological control or biocontrol is scientifically attractive goal, albeit often not as commercially successful in most cases compared to chemical control methods. It has become increasingly attractive since methyl bromide has been proven harmful to the environment, livestock and humans and subsequently banned from use (Duniway, 2002). Although there are several chemical alternatives to methyl bromide available in the market today, their mechanisms are either poorly understood or not extremely specific to the target nematodes. Furthermore, biocontrol also offers the option to isolate bioactive metabolites from microorganisms and develop them into bionematicides, in addition to using the organism by itself. It is also a good idea to develop biocontrol agents and bionematicides to work synergistically with currently employed practices such as genetic resistance, crop rotation and other chemical nematicides as well, for a well-rounded, integrated nematode management.

Several microorganisms have been tested as potential antagonists against SCN, including both fungi and bacteria, with *Pasteuria* spp. being the most important bacterial agent. However, only a few bacterial and fungal biocontrol agents have been commercialized thus far, mostly in the genera *Bacillus* or *Pasteuria* for bacteria, and *Paecilomyces* and *Pochonia* for fungi (Li et al., 2015; Chen and Dickson, 2012). Bionematicides of bacterial origin, especially Abemectin, have seen some commercial success, including for use on *Heterodera avenae* (Zhang et al., 2017). However, eukaryotic fungi and other fungal-like organisms that can sporulate, have only been increasingly reported as potential biocontrol agents, especially on root-knot nematodes, but have had fewer commercial successes for SCN (Tranier et al., 2014; Musil, 2016). Filamentous fungi could overwinter, produce spores and remain in the soil for longer periods of time than their non-sporulating counterparts (Jung et al., 2014); and thus, may reduce reapplication rates and associated costs. Given that the major soybean producing states (Iowa, Illinois, Minnesota, the Dakotas and Ohio) (USDA, 2016) have harsh winters with fallow fields and conditions unfavorable for any growth, filamentous and sporulating fungi could be the most viable option for biocontrol, yet few have been commercially developed. Furthermore, desiccated spores enable easy storage and transport of the organism without losing viability for long periods compared to live vegetative cells. Desiccated fungal spores could also be easily formulated to be applied as soil drenches like in the case of Melocon WG or coated on seeds as in the case of Poncho/Votivo (BAYER, 2012, 2015).

There are several reviews that discuss biological control of several plant-parasitic nematodes using fungi. This review aims to specifically summarize studies that have tried to understand the fungal communities associated with one nematode, SCN, in the USA. A detailed review of biocontrol of all plant-parasitic nematodes by different kinds of fungal parasites such as nematode-trapping fungi, obligate endoparasites of SCN juveniles, and female and egg parasites, on different stages of nematode development, is presented in a book chapter by Chen and Dickson (2012). Therefore, this review will focus mainly on members of culturable fungal communities from SCN that can be studied *in vitro* as well as *in vivo* for their biocontrol properties. For a good biological control agent, in addition to being able to colonize nematodes *in vitro,* the ability to tolerate abiotic and biotic stresses to survive in soil or colonize roots is an important consideration. Fungistasis, or competition between fungi, as well as host preferences (Mauchline et al., 2004) and abiotic stresses such as varying conditions of temperature, humidity, pH and moisture in the soil environment must be evaluated before a biocontrol agent is tested in the field. Several reviews have discussed the role of nematode-trapping fungi that utilize various trapping devices, such as nets, rings and knobs, in control of nematodes (Mankau, 1980; Kerry, 1988; Siddiqui and Mahmood, 1996; Chen and Dickson, 2012). This review will not discuss trapping fungi as they often are influenced by other soil organisms and competition, and are often fastidious to culture (Persmark et al., 1996; Jaffee et al., 1998; Li et al., 2011). This paper aims to summarize some of the common fungal taxa reported in the literature, associated with SCN, as well as anti-nemic metabolites from fungi that have been studied for their potential against SCN and other cyst nematodes. This review also discusses various soil amendments that have been shown to reduce SCN levels in the soil, which may have indirect effects on fungal communities in the soil affecting SCN.

## Common culturable fungi (mycobiome) associated with SCN cysts and eggs

The diversity of fungi and fungal-like organisms is vast, and soil is a very complex habitat that hosts all kinds of fungi with varying modes of nutrition. Obligate parasites of SCN are highly virulent and effective nematode parasites, but they have limitations as viable biocontrol options as they cannot be cultured easily on artificial media and other fungi usually outcompete them in soil. On the other end of the continuum, true saprophytes can be easily cultured on artificial media, but generally may not colonize the SCN as pathogens. Thus, facultative saprophytes that are both effective parasites of nematodes, but can also be cultured on artificial media have promise as potential biocontrol agents.

**Figure 1:**
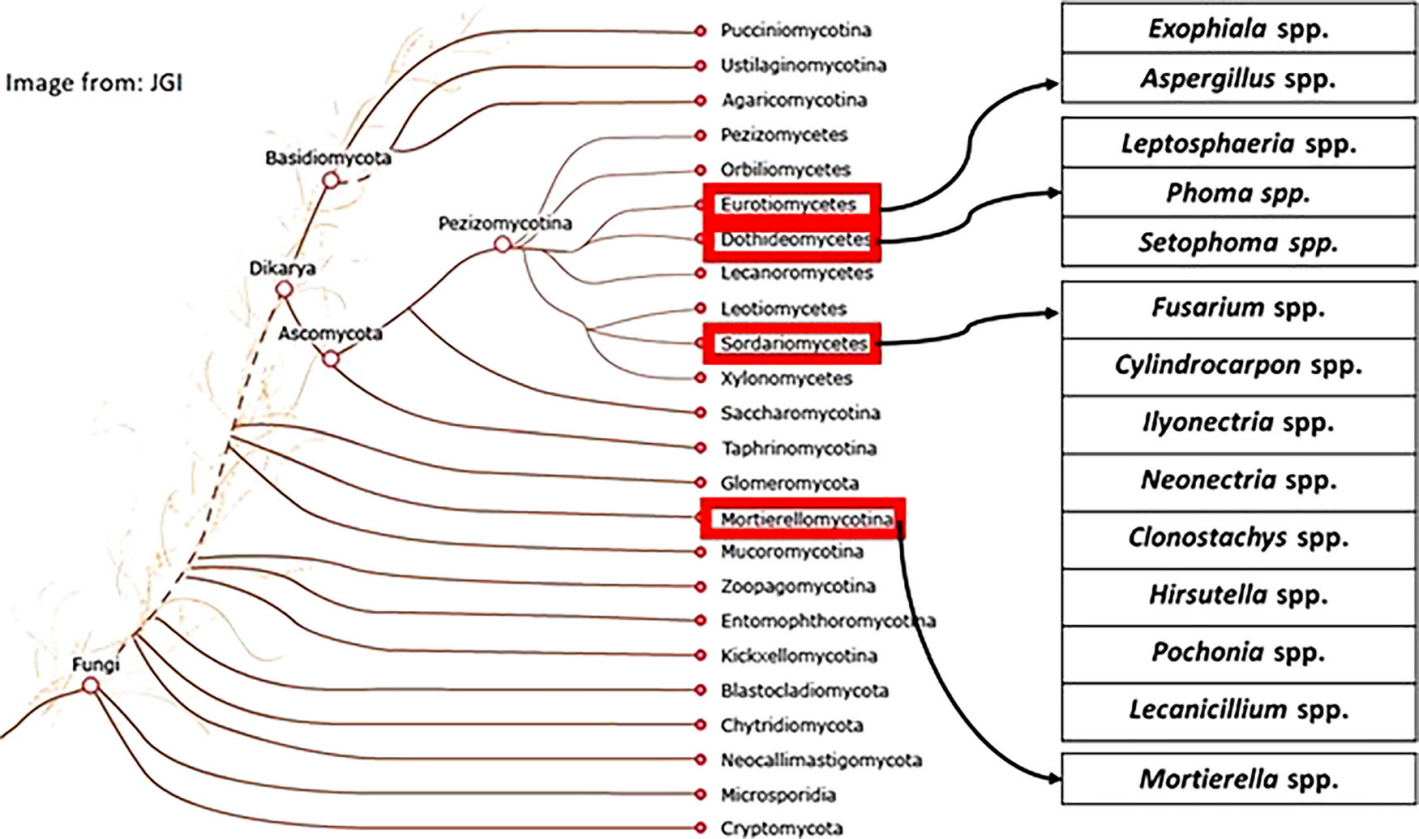
Common fungi isolated from SCN cysts (US SCN mycobiome studies) and their relationship within the Kingdom Fungi. The image was obtained from the Joint genome Institute (JGI) fungal program and MycoCosm and edited to suit this manuscript.

**Figure 2:**
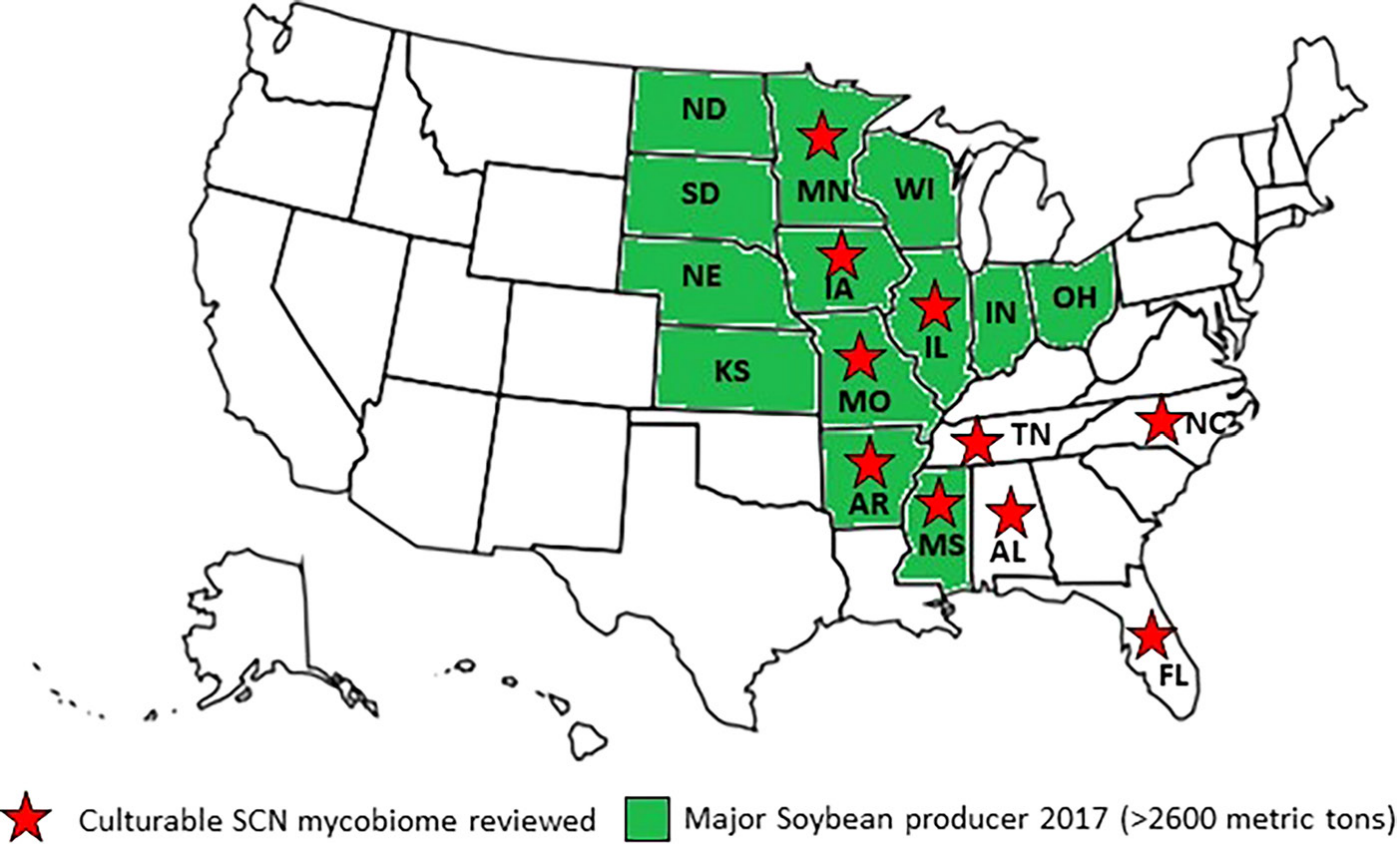
Top soybean producing states (colored green) and locations of studies where SCN mycobiome was analyzed (red stars).

Several studies have investigated the fungi associated with the SCN from several locations of the USA. Figure 1 indicates the major fungal genera isolated from SCN cysts (culturable mycobiome studies from the USA) on a fungal tree generated by the Joint Genome Institute fungal program and the MycoCosm initiative (Grigoriev et al., 2011, 2014). Figure 2 indicates major soybean producing states in the USA (Economic Research Service USDA, 2017) and locations from where SCN cyst mycobiomes have been isolated, analyzed, and summarized in this report. Most often, studies that report potential biocontrol fungi seek natural parasites and hence culture fungi from cysts or females or eggs or J2 of SCN. *Heterodera glycines* cyst, female and egg parasites are relatively easy to locate and isolate from the environment as they are the sedentary overwintering structures in the soil. It would be practical and beneficial to target the immobile and resistant life stages, which are survival structures that allow the hundreds of eggs in each cyst to survive dormant for years in soil.

### Family Nectriaceae: *Fusarium*, *Cylindrocarpon* and other genera

Assessment of fungal genera associated with SCN cysts and eggs as potential biocontrol agents have been reported for more than three decades. These studies have reported *Fusarium* spp., to be highly associated with SCN cysts, the most common species being *F. oxysporum* and *F. solani.* However, using molecular techniques for species identification, we know now that these groups form large species complexes that may harbor cryptic species and are highly diverse (O’Donnell et al., 2008, 2009; Muraosa et al., 2014).

Several studies have examined the culturable mycobiome or the collection of culturable fungi from SCN in the USA. A study published from Alabama by Gintis et al. (1983) looking at fungal diversity associated with several stages of cysts, ranging from the cream-colored females attached to the root tissue to the brown cysts encasing eggs that are dispersed into soil, provided insights into the importance of the genus *Fusarium*. *Fusarium oxysporum* and *F. solani* are omnipresent soil fungi that help degrade residual plant materials. Fungi in these large species complexes have highly diverse lifestyles. There are several members in this group of fungi that are specific pathogens of plants, animals and even humans (Rajendran and Ramakrishnan, 2015; Zhang et al., 2006, 2007) while there are those that are saprophytes in the soil and could become pathogenic when proper plant hosts are present, and some endophytes that could increase fitness of plants against pathogens such as nematodes (Banihashemi and Dezeeuw, 1973; Waweru et al., 2014). Some may also colonize SCN cysts or produce metabolites that hinder the lifecycle of the nematode. In this study (Gintis et al., 1983), *Fusarium* spp. were isolated at a very high frequency from all stages of SCN cysts and the authors also discuss that several other studies prior to this publication found similar results. For example, in a similar study conducted on cream-colored females that were still attached to the soybean roots, from four different locations in North Carolina, 31% of all fungi isolated were *Fusarium* spp. (Gintis et al., 1982). Similarly, *Fusarium* spp., especially *F. oxysporum* and *F. solani* have been reported as the most prevalent fungi associated with SCN cysts in a study in two soybean fields in Illinois (Carris et al., 1989). Over a period of three years, this study observed a high frequency of *Fusarium* and closely related fungi within Nectriaceae from both locations. In fact, both studies reported that anywhere between 30 to 60% of all fungi isolated from the cysts belonged to *Fusarium* or other allied genera such as *Cylindrocarpon* or *Neocosmospora*. An older and broader study done across the southern states of Arkansas, Florida, Mississippi and Missouri (Morgan-Jones et al., 1981), in which 250 cysts from one field from each state were studied, also reports to have observed more *Fusarium* than any other genera. Nearly 38% of all cyst fungi from Arkansas, 61.5% from Florida, 50% from Missouri and 52% from Mississippi locations were either *F. oxysporum* or *F. solani*. Chen et al. (1994) also reported a high frequency of this genus from soybean fields in Florida and almost 37% of all fungi isolated from SCN cysts in Tennessee fields were also from the genus *Fusarium*. While examining the effects of various management practices on the SCN mycobiome, Bernard et al. (1996) also discovered that *Fusarium* spp. were the most frequently isolated from SCN females from both the field and from roots grown in greenhouse. Chen and Chen (2002) conducted a large state-wide study in Minnesota involving 4,500 SCN cysts, 4,500 females and 45,000 eggs across 45 fields from 26 counties. Their reports also indicate similar trends. It was also noted by Gintis et al. (1983) that continuous cropping of soybean or soybean monoculture increased the frequency of *F. oxysporum* and *F. solani.* Species of *Fusarium* are strongly associated with SCN cysts. In our recent study done in Minnesota, on a long-term soy-corn rotation system, *Fusarium* was the most frequently isolated genus, with about 30% of all fungi isolated from 6,000 mature SCN cysts (Haarith et al., 2019). Although further *in vitro* testing is needed to determine their virulence and ability to parasitize and kill SCN eggs within cysts, one cannot overlook their possible potential important role in biocontrol of SCN, given their abundance and taxonomic complexity.

Beyond the genus *Fusarium*, other fungi from the family Nectriaceae appear to also be common inhabitants of SCN cysts. *Cylindrocarpon destructans* and *Cylindrocarpon olidum* are two other fungi that have been reported in these studies (Morgan-Jones et al., 1981; Gintis et al., 1982, 1983; Carris et al., 1989; Chen et al., 1994) to be commonly isolated from SCN. There have also been reports of *Cylindrocarpon* spp., found associated with potato cyst nematodes (PCN) in the tropics (Rodriguez-Kabana and Morgan-Jones, 1988). Many Nectriaceae fungi are morphologically similar. Hence, many of the earlier studies based only on morphology may not have resolved the different Nectriaceae genera. Sterile fungi that do not produce any spores are difficult to identify morphologically. Hence, there is a need for molecular studies to carefully dissect and better characterize the taxonomy of this group, in addition to morphological characterization. In our culturable SCN cyst mycobiome (Haarith et al. 2019), of the top 14 genera that made up about 80% of all fungi isolated, Nectriaceae other than *Fusarium* spp. such as *Ilyonectria* spp., *Cylindrocarpon* spp., and *Neonectria* spp. contributed to about 10% of all fungi isolated. Therefore, the net contribution of this fungal family to the mycobiome was about 40%.

### Family Clavicipitaceae: *Pochonia*

All the previously discussed regional studies on SCN cyst fungal diversity that reported high frequency of *Fusarium* spp. also found several members of closely related families within the order Hypocreales, including Cordycipitaceae and Clavicipitaceae. The most common fungus from Clavicipitaceae isolated from cysts is *Pochonia chlamydosporia* (syn. *Verticillium chlamydosporium, Metacordyceps chlamydosporia*, *Cordyceps chlamydosporia, Diheterospora chlamydosporia*). Many other studies have shown *P. chlamydosporia* to have potential to antagonize SCN. Interestingly, it was Gintis et al. (1983) who first reported the relationship of *P. chlamydosporia* with SCN cysts although its association with the genus *Heterodera* was previously reported by Tribe (1977), who hypothesized it was a potential nematode pathogen. The pioneering work of Kerry et al. (1982) showed that *P. chlamydosporia* was able to control the cereal cyst nematode. Morgan-Jones et al. (1983) showed that this fungus directly inhibited the hatching of *Meloidogyne arenaria* (root-knot nematode) by colonization and hyphal penetration. In a study conducted on sugar beet cyst nematode *Heterodera schachtii*, Crump (1991) and Crump and Irving (1992) observed 75% biocontrol while Muller (1982) and Siddiqui and Mahmood (1995) reported a loss of reproductive capacity of *H. schachtii* due to *P. chlamydosporia.* The loss of female fecundity over a period will ensure a significant reduction in the number of cysts and eggs that could cause disease either later in the season or in the following year. *Pochonia chlamydosporia* was also frequently isolated (5%) from eggs in a study examining fungal parasitism of SCN in different cropping-tillage regime (Bernard et al., 1996). Although no successful field trials of *P. chlamydosporia* have been reported in the United States against SCN, at the time of authoring this manuscript, Tobin et al. (2008) from the UK reported success in controlling PCN (*Globodera pallida* and *G. rostochiensis*) in the field using *P. chlamydosporia.* In our SCN cyst mycobiome study, *Pochonia* spp. contributed to about 4% of all fungi isolated (Haarith et al., 2019). A recent review by Manzanilla-Lopez et al. (2013) discusses both toxicity and parasitism exhibited by this fungus as well as the many efforts that are underway to test the durability of this fungus in real agricultural environments and the practical issues of implementing *P. chlamydosporia* as a biocontrol agent.

### Family Cordycipitaceae: *Lecanicillium*

Previously known as *Verticillium lecanii, Lecanicillium lecanii* has also shown promise as a potential biocontrol agent. It is noteworthy that classification based on morphology previously placed the two fungi, *P. chlamydosporia* and *L. lecanii*, in the genus *Verticillium*, while recent molecular analyses clearly showed that *Verticillium* is polyphyletic, and places these fungi in different families, Clavicipitaceae and Cordicypitaceae, respectively (Sung et al., 2007). This further emphasizes the need for molecular studies to identify natural fungal parasites of SCN eggs. Recent rDNA evaluations have separated the entomopathogenic strains of *V. lecanii* into a separate genus (*Lecanicillium)* and resolved it into several species (Koike et al., 2011), each with differing abilities to colonize SCN. Owing to its ability to colonize insects, *Lecanicillium* spp. are not new to the biocontrol literature. A strain of *L. lecanii* isolated from SCN was studied for its ability to colonize various nematode structures. Microscopic evaluations revealed that within 16 hr of incubation, the fungus colonized SCN females, cysts, eggs and the gelatinous matrix (Meyer and Wergin, 1998). Several species of *Lecanicillium* have been used to control aphids and other insects as well as plant pathogenic fungi (Faria and Wraight, 2007; Kim et al., 2010). Protoplast fusion using nitrate non-utilizing (Nit) mutants of commercially available *L. muscarium*, *L. longisporium* and other *L. muscarium* strains capable of controlling insects and other phytopathogens were used to attempt to create a super-agent that could control insects, SCN as well as some phytopathogenic fungi (Koike et al., 2011; Shinya et al., 2008b, 2008a). Their findings indicated that many of these isolates had high toxicity effects on nematode eggs and culture filtrates from those strains produced visible developmental damage in SCN eggs. They also report the loss of female fecundity and direct parasitism of SCN eggs by *Lecanicillium* spp. As these fungi possess more than one mode of action, such organisms are good candidates for biocontrol.

### Family Ophiocordycipitaceae: *Hirsutella* and *Purpureocillium*

*Hirsutella* spp. are one of the most discussed group of fungi with respect to biological control of SCN, with new species being recently discovered in Minnesota (Chen et al., 2000; Chen and Liu, 2007; Liu and Chen, 2005). *Hirsutella rhossiliensis* and *H. minnesotensis* are two fungi that have been found to possess promising biocontrol abilities in a greenhouse study (Chen and Liu, 2005). Greenhouse studies of its ability to control SCN numbers were done alongside development of specific PCR based quantification methods for detecting this fungus in soil (Zhang et al., 2006, 2008). Unlike *Fusarium* or other Nectriaceae discussed earlier, *Hirsutella* spp. are not egg parasites, but are obligate parasites of J2 and other vermiform motile stages. In a study evaluating suppression of SCN in long-term soybean monoculture, two different fields were compared with each other and with a soybean-corn rotation plot. *Hirsutella rhossiliensis* was observed to have parasitized most of the J2s in this field (Chen, 2007). However, as discussed earlier and in other reports, obligate parasites such as *Hirsutella* pose challenges for development of biocontrol strategies (Chen, 2004; Jaffee and Zehr, 1985) and commercial development as they are slow growing and have limited saprophytic abilities.

Many reviews to date have discussed the presence of *Purpureocillium lilacinum*, named based on its ability to produce purple spores, as a key component in nematode suppressive soils (Sun and Liu 2000). It is a commonly occurring natural soil fungus, which was initially identified using morphology as *Paecilomyces lilacinus*, and was later given its own genus (*Purpureocillium*) within the family Ophiocordycipitaceae, based on molecular analyses showing that *Paecilomyces* is a polyphyletic genus (one genus represented in several phylogenetic clades), and its medical importance as an opportunistic animal and human pathogen (Luangsa-Ard et al., 2011; de Sequeira et al., 2017; Demitsu et al., 2017; Trinh and Angarone, 2017; Sung et al., 2007). However, many agronomically important strains of this fungus are not necessarily animal or human pathogens. In fact, *P. lilacinum* YES-2 has been studied for biological control of plant nematodes, while strain QLP12 has been used against fungal pathogens (Lan et al., 2017; Aminuzzaman et al., 2013). Unlike *Hirsutella* spp. it has been found primarily associated with nematode eggs (Mwaheb et al., 2017).

### Family Herpotrichiellaceae: *Exophiala*

The genus *Exophiala* can be traced back to 1980s and beyond, as a commonly isolated natural antagonist to SCN (Wrather and Anand, 1984). In all the studies that have been discussed in this review focused on identifying fungal communities associated with SCN cysts, *Exophiala* spp., have been consistently reported. However, it has been isolated with far lower frequency than that of *Fusarium* spp., *Cylindrocarpon* spp., *M. chlamydosporia* or *Lecanicillium* spp. It would be a valuable insight to evaluate the role of this genus in SCN infested soils. Some of the common species identified with SCN were *E. equina*, *E. salmonis* and *E. pisciphila*, but *E. oligosperma* has been found on free-living marine nematodes (Bhadury et al., 2009). *Exophiala pisciphila* was shown moderately pathogenic to SCN eggs (Chen et al., 1996). *Exophiala* spp. contributed to about 5% of the culturable SCN cyst mycobiome in our recent study (Haarith et al., 2019). Further studies are needed to determine their role in natural control of nematodes and potential of any species and isolates as commercial biocontrol agents.

A summary of all the common fungi in SCN mycobiomes are listed in Table 1. It is evident that there are several common genera of fungi that have been isolated across all these diverse mycobiome studies done in the USA. It is possible that both the root exudates of soybean plants as well as the niche microcosm of SCN as an organism could both influence the selection of only a few groups of fungi to be associated with SCN (Haarith et al., 2019).

**Table 1.** Common fungi isolated from SCN mycobiome studies in the United States.

Fungal class		Fungal family		Fungal genus		Isolated from				References	
Females	Cysts	Eggs	Juveniles
Sordariomycetes	Nectriaceae	*Fusarium*	Yes	Yes	Yes		(Morgan-Jones et al., 1981; Gintis et al., 1982, 1983; Carris et al., 1989; Chen et al., 1994; Bernard et al., 1996; Chen and Chen, 2002; Haarith et al., 2019)
		*Cylindrocarpon*		Yes	Yes		(Carris et al., 1989; Chen and Chen, 2002; Haarith et al., 2019)
		*Neocosmospora*	Yes	Yes			(Morgan-Jones et al., 1981; Gintis et al., 1982, 1983; Carris et al., 1989; Chen et al., 1994; Bernard et al., 1996)
		*Ilyonectria*		Yes			(Carris et al., 1989)
		*Neonectria*		Yes			(Haarith et al., 2019)
	Bionectriaceae	*Clonostachys*		Yes			(Haarith et al., 2019)
	Clavicipitaceae	*Pochonia*	Yes	Yes			(Gintis et al., 1983; Carris et al., 1989; Bernard et al., 1996; Haarith et al., 2019)
	Cordycypitaceae	*Lecanicillium*	Yes	Yes			(Gintis et al., 1982, 1983; Carris et al., 1989; Chen et al., 1994; Haarith et al., 2019)
	Ophiocordycipitaceae	*Hirsutella*				Yes	(Chen et al., 2000; Chen and Liu, 2005, 2007; Liu and Chen, 2005)
		*Purpureocillium*	Yes	Yes			(Gintis et al., 1983; Carris et al., 1989; Bernard et al., 1996; Haarith et al., 2019)
Eurotiomycetes	Herpotrichiallaceae	*Exophiala*	Yes	Yes	Yes		(Morgan-Jones et al., 1981; Gintis et al., 1982, 1983; Carris et al., 1989; Chen et al., 1994; Chen and Chen, 2002; Haarith et al., 2019)
Dothidiomycetes	Leptosphaeraceae	*Leptosphaeria*		Yes			(Chen and Chen, 2002; Haarith et al., 2019)
	Didymellaceae	*Phoma*	Yes	Yes			(Morgan-Jones et al., 1981; Gintis et al., 1982, 1983; Carris et al., 1989; Chen et al., 1994; Bernard et al., 1996; Haarith et al., 2019)
	Phaeosphaeriaceae	*Setophoma*		Yes			(Haarith et al., 2019)
Mortierellomycetes	Mortierellaceae	*Mortierella*		Yes			(Haarith et al., 2019)

## Fungal metabolites in fungal–SCN interactions

We have discussed fungi that can colonize eggs and other stages of nematodes and feed off several nematode stages. As lysotrophic organisms, fungi rely on the production of extracellular metabolites and enzymes to externally breakdown substrates into simpler organic molecules before assimilating them (Richards and Talbot, 2018). Some fungi also make specialized secondary metabolites for fitness advantages in specific ecological niches (Fox and Howlett, 2008). Acids, alkaloids, terpenes, enzymes and antibiotics are some of the commonly produced fungal metabolites that have been cited to be involved in fungal interactions with nematodes (Huang et al., 2015; Dihingia et al., 2017). Most of what is known about fungal–nematode interactions at the molecular level come from studies of the model organism *Caenorhabditis elegans*, not the SCN (Li et al., 2015).

In several studies, fungi have been isolated from SCN cysts and eggs, identified, and tested for the effects of their culture filtrates on nematode morphology and physiology. The ability of fungi to produce metabolites toxic to SCN or other nematodes in submerged liquid culture or on solid substrates such as rice medium in the laboratory might not mirror the natural environment in soil. However, it is useful to identify compounds that have high toxicity and specificity toward SCN and other nematodes which are also: safe to other soil inhabitants, animals and humans; cheap and easy to synthesize; and biodegradable. These could potentially be useful biopesticides in agriculture or as pharmaceuticals.

The genus *Fusarium* is currently being explored for its potential to produce secondary metabolites and toxins. In a recent study, culture filtrates of *Fusarium oxysporum* strain 162 were analyzed for nematicidal activity (Bogner et al., 2016). The fungus was cultured on rice media and then extracted with ethanol. Several fractions were obtained using other organic solvents and chromatography techniques. Six new compounds were reported for the first time from *Fusarium.* Four compounds were found to be significantly detrimental to SCN and RKN and one, 4-hydroxybenzoic acid, was found to be almost as effective in killing nematodes as the nematicide carbofuran (Bogner et al., 2016). The same fungus was first isolated in Kenya as a tomato endophyte and several groups from China and Europe have since investigated its potential as a nematode biocontrol agent. Endophytes can be beneficial to plants against pests like SCN both directly and indirectly (Bajaj et al., 2015; Daneshkhah et al., 2013). In fact, this particular strain of *F. oxysporum* was subjected to submerged fermentation and the culture broth was tested on a diverse array of nematodes belonging to different ecological niches and was found to have negative effects on almost all of them except for fungivores (Hallmann and Sikora, 1996). This study used a gliotoxin-inducing fermentation medium, specifically designed to induce mycotoxins, and found 20 of their 34 culture filtrates to be effective against nematodes.

Similarly, a study of a strain of *F. equiseti* isolated from SCN was grown in potato dextrose agar and the entire petri dish of media and fungus were homogenized into potato dextrose broth, subjecting the strain to both solid-substrate and submerged fermentation. Culture filtrates inhibited SCN hatch significantly when compared to water and uninoculated potato dextrose broth media (Nitao et al., 1999). The study used four other media (yeast-lactose, cornmeal, soybean and V8 juice) as hatch controls and deduced that all microbial growth media inhibited hatch when compared to water, even without fungi growing in them (Nitao et al., 1999). In another study, when SCN J2s were exposed to culture filtrates of native and culturable SCN mycobiome, *Paecilomyces lilacinus* and *Staganospora* spp. were found most effective, while *F. oxysporum*, *E. pisciphila*, *Gliocladium catenulatum* and *Pyrenochaeta* spp. culture filtrates did not produce detectable effects. Furthermore, the study also tested two kinds of media, Czepek-Dox broth and malt extract broth, the latter being more effective in inducing metabolite production (Chen et al., 2000). Comparing the two media, Czepek-Dox broth has 30 g/L sucrose as a carbon source while Malt Extract broth has only 17 g/L of carbon in the form of malt extract. Carbon starvation is often reported as a condition necessary to initiate secondary metabolism (Stappler et al., 2016).

Analysis of a known SCN infested field in China, conducted by USDA-ARS, resulted in a mycobiome of 253 isolates belonging to 17 different genera and 23 species. Only 9.1% of all these isolates produced culture filtrates that significantly inhibited SCN hatch (Meyer et al., 2004). All *Cylindrocarpon* strains and 25% of all *Fusarium* strains exhibited significant decrease in hatch while 6% of *Fusarium* strains and about 8% of *Pochonia* strains stimulated hatch. Interestingly, the study also measured pH of the culture filtrates and found no significant differences between those that inhibited and stimulated SCN egg hatch. It is noteworthy that the composition of mycobiome in this Chinese soybean field was not very different from those isolated in the USA discussed previously.

Linoleic acid and several other chlorinated aliphatic compounds from *Lachnum papyraceum* were reported to have nematicidal and even anti-microbial properties, therefore supporting the idea that soil microbial communities are interdependent and strongly interacting (Anke et al., 1995). One compound may have multiple effects on several soil inhabitants. This makes *in vivo* testing compulsory to screen for good biocontrol agents. Apart from beauvericin and enniatins, there are many new toxins being isolated from *Fusarium* spp., termed emerging fusaritoxins, such as fusaproliferin, and moniliformins (Jestoi, 2008). It will be informative to test some of these toxins against SCN, as a majority of the SCN mycobiome appears to be composed of *Fusarium* spp. Some *Fusarium* metabolites have been tested against nematodes other than the SCN and found to be effective bionematicides. At 100 μg/mL concentration *in vitro*, bikaverin and fusaric acid were able to kill about 50% of all of pinewood nematode treated over a two day period (Kwon et al., 2007). Flavipin, isolated from the culture broth of a *Chaetomium globosum* strain, were also shown to produce anti-nemic compounds in culture. Culture broth was screened for ability to curb egg hatch and mobility of both SCN and RKN and subsequently flavipin was purified and identified using nuclear magnetic resonance spectroscopy to be the active ingredient. This pilot study revealed that the compound had good results *in vitro* while greenhouse assays showed no difference from the control as bioavailability of this compound in soil environment needs to be investigated (Nitao et al., 2002).

The J2 is the most exposed life stage of the SCN to soil microbes, as they move through soil toward soybean roots to establish a feeding site. Until they molt for the first time and subsequently hatch out of eggs, J2s are protected by the chitinous and glycoprotein rich eggs, which are in turn encased within the melanized SCN female body or cyst (Hirshmann 1981). Nematode cuticle is very similar to animal skin as it is primarily made up of collagen and other glycoproteins, while the shell of eggs is rich in chitin (Cox et al., 1981; Niblack et al., 2006). Therefore, an egg-parasitic fungus needs to produce chitinases, collagenases and carboxypeptidases while J2 parasites would primarily have to produce collagenases, other carboxypeptidases and Carbohydrate-Active enZymes (CAZymes) (Larriba et al., 2014; Page et al., 2014). Some predatory fungi also produce small molecule acids that are detrimental to nematodes (Anke et al., 1995; Stadler et al., 1993). Similar tactics are used by egg and J2 parasites as well.

Secreted fungal enzymes with the ability to breakdown cuticle or the outer layer of eggs or antagonize plant-parasitic nematodes also have a role in control of nematodes, including SCN. The first serine protease from a nematode parasitic fungus was identified and reported from *Pochonia rubescens*, (Lopez-Llorca, 1990). Since then, several extracellular serine proteases from nematode parasitic fungi have been purified and in some cases, the genes involved in their synthesis have been cloned (Yang et al., 2007; Tunlid et al., 2017). Serine proteases can help degrade nematode cuticle, thereby giving fungi access to the nematode body to derive nutrition (Zhang et al., 2008; Tunlid and Jansson, 1991; Tunlid et al., 2017). *Hirsutella rhossiliensis* is well known as an obligate SCN J2 parasite (Liu and Chen, 2000, 2001). A thermostable serine protease from *H. rhossiliensis* was recently isolated and purified, and was also proven to have nematicidal activity against SCN (Wang et al., 2007). Similarly, upon analysis of the genome of *Purpureocillium lilacinum*, a close relative of *Hirsutella* spp., several CAZymes, 13 polyketide synthase clusters, 10 non-ribosomal peptide synthase clusters, and several other genes or clusters involved in production of secondary metabolites were found, including one responsible for producing the antibiotic Leucinostatin (Wang et al., 2016). The proteinase VCP1 from *Pochonia chlamydosporia* is shown to be highly host specific as its activity on *Meloidogyne incognita* eggs was significantly better than on those of *Globodera rostochiensis* (Segers et al., 1996). Whole genome sequencing and comparative genomics could also greatly accelerate discoveries of new nematicidal compounds (Prasad et al., 2015).

The first chitinase shown to have activity against nematodes, Chi43, was also purified from the genus *Pochonia* (Tikhonov et al., 2002). As discussed earlier, chitinases help fungi weaken the nematode eggshell but are mainly useful to fungi in terms of hyphal growth. The endochitinase was purified from *V. chlamydosporium* (now *Pochonia chlamydosporia*) and *V. suchalasporium* grown in submerged fermentation in a semi-liquid, colloidal chitin medium for about 20 days. Microscopic evaluations of the effect of this enzyme of PCN, a close relative of SCN, revealed peeling of eggshells post exposure (Tikhonov et al., 2002).

A summary of this metabolite discussion is presented in Table 2. Although there are several small molecules, organic acids and enzymes isolated and proven to be antagonistic to SCN *in vitro*, they must be evaluated *in vivo* before they could become potential bionematicides. Greenhouse studies on bioavailability and specificity, followed by *in vitro* studies of human and animal toxicity of these compounds are important next steps, and are yet to be reported.

**Table 2.** Specific compounds characterized from common fungi discussed in this review.

Fungal origin	Fungal compound	Class of compound	References
*Chaetomium* sp.	Flavipin	Aryl hydrocarbon	(Nitao et al., 2002)
*Fusarium* spp.	4-hydroxy benzoic acid	Organic acid	(Bogner et al., 2016)
	Bikaverin	Organic heterocyclic hydrocarbon	(Kwon et al., 2007)
	Fusaric acid	Organic acid	(Kwon et al., 2007)
*Lachnum* sp.	Linoleic acid	Organic acid	(Anke et al., 1995)
*Pochonia* spp.	Serine proteases	Protease enzyme	(Lopez-Llorca, 1990; Segers et al., 1996; Wang et al., 2007)
	Chitinase	Chitinase enzyme	(Tikhonov et al., 2002)
*Purpureocillium* sp.	Leucinostatin	CAZ-yme	(Wang et al., 2016)

## Indirect fungal biocontrol through soil amendments

Crop rotation with non-host crops, such as corn and reduced tillage, have been reported as viable methods for SCN control (Melakeberhan et al., 2015). The ability of non-host crops to reduce the nematode population densities stems from the inability of SCN to invade or establish feeding sites in non-hosts and the subsequent starvation of the nematode to death, but also could be due to a change in the soil microbiome through changes in plant root exudates and plant residues. Addition of such exudates and botanical compounds to the soil, therefore, is also a way to promote biological control of SCN or any other nematode (Riga et al., 2001). There are many routes by which organic amendments to soil can potentially be detrimental to SCN, apart from crop rotation (Chitwood, 2002; Oka, 2010). One of the ways cover crops and non-host rotation could reduce nematode population densities is by favoring nematode antagonistic fungi in the soil environment (Oka, 2010). In a recent metabarcoding study observing the mycobiome of a long-term soy-corn rotation experiment, Hu et al. (2018) noted that SCN egg densities (eggs/100 cm^3^ of soil) reduced in fields where soybean were interspersed with five years of corn. This was correlated with a change in species richness and rhizosphere beta diversity, possibly explained by the effects of root exudates and crop residues primarily.

Crop rotation with corn will not be favorable economically if corn prices decline considerably while there is no change or a decline in the global demands and prices of soybean. Instead, pre-planting with cover crops such as sunn hemp (*Crotalaria juncea*) have been studied and found effective in lowering nematode inoculum levels, apart from symbiotic relationships with nitrogen fixing bacteria (Wang et al., 2002). In a greenhouse study, sunn hemp is one of the most effective rotation crops in lowering SCN population density (Warnke et al., 2006). *Crotalaria striata* was especially found to be effective in controlling SCN (Valle et al., 1995). Several other cover crops have also been suggested and tested. In recent studies of African indigenous vegetables such as African nightshades (*Solanum villosum* and *S. scabrum*) and African spinach (*Amaranthus dubius* and *A. cruentus*), both field surveys and field trials, the nightshades (non-tuberous Solanaceae plants) significantly reduced the PCN population when used as a cover crop before growing potatoes (Chitambo et al., 2019). In the same study, in addition to the absence of any cysts on the roots of the nightshade, egg viability of cysts isolated from the rhizosphere of nightshade were shown to have reduced viability. In another study exploring cover crops, Brassicaceae such as brown mustard (*Brassica juncea*) and winter canola and winter rapeseed (*B. napus*) and field rye (*Secale cereal*) reduced several important bacterial and fungal soybean diseases including foliar diseases such as septoria leaf blight and root diseases such as sudden death syndrome, indicating the indirect manipulation of the microbial community in that soil (Wen et al., 2017). The study also reported significant reduction of SCN eggs post field rye and rapeseed cover cropping.

Although changing crops and using cover crops changes the phytobiome, there is evidence that monoculture may be more effective at controlling SCN numbers by aiding accumulation of natural fungal antagonists in the soil. When 10% of suppressive soil from three long-term soybean monoculture (five or more years) fields in China were mixed individually with 90% sterile soils, all three mixtures still retained its ability to suppress SCN compared to controls (Sun and Liu, 2000). The same study observed an abundance of *P. lilacinum* in all three suppressive soils. Similarly, the accumulation of *H. rhossiliensis* in these soils was attributed to soil suppression of SCN, from evaluating long-term monoculture soil in potted-plant experiments in a greenhouse in Minnesota (Chen, 2007). An increase in rhizosphere *Purpureocillium lilacinum* and *Pochonia chlamydosporia*, along with some Pseudomonad bacteria, was observed and negatively correlated with SCN numbers in soil from several long-term soybean monoculture plots (Hamid et al., 2017).

Certain polymers such as chitosan have been shown to affect fungi and plants alike (Lopez-Moya et al., 2019). The nematode egg parasite *Pochonia chlamydosporia* was seen to have increased protease production and appressorial differentiation in the presence of chitosan, thereby being more efficient in parasitizing nematode eggs (Escudero et al., 2016). Similarly, chitin from crustacean shells, a polymer from which chitosan is produced by deacetylation, also increased certain fungal groups such as *Lecanicillium lecanii* and *Geotrichum candidum* when added to soil between 0.5 and 4% (w/w) (Rodríguez-Kábana et al., 1984). The same study also noted an overall increase in the amount of fungi and actinobacteria post chitin amendment. Manure and domestic wastes have also been studied for their ability to control SCN in the fields (Bao et al., 2013; Reynolds et al., 1999; Yeates et al., 2006). Digested swine manure with enriched concentrations of ammonia and volatile fatty acids were tested and found to heavily inhibit SCN egg hatch, as well as be lethal to hatchlings or juveniles, reducing their ability to infect soybean seedlings (Xiao et al., 2008). Similarly, a combination of cow manure and chemical fertilizer or simply the addition of fertilizer alone altered the nematode community structure in an organic continuous soybean monoculture field, reducing the population of parasitic nematodes (Li et al., 2018). A simple explanation for these observations might be the change in the carbon:nitrogen ratio in the soil environment, which further influences the soil microbial community structure. Therefore, one cannot discount the possibility of these amendments directly and positively supporting fungal communities in the soil that are also natural antagonists to SCN, in addition to any possible directly toxic effects on SCN itself.

Although such studies are extremely difficult to accomplish, there have been some studies that have tracked the decrease of nematodes correlated with addition of organic matter to the soil as well as an increase in fungal groups, such as *Trichoderma* spp., *Penicillium* spp. and *Aspergillus* spp., which have all been implicated in nematode biocontrol (Hoitink and Boehm, 1999; Hoitink et al., 1997) and have been isolated from SCN cysts as detailed in the previous sections. In another study, *P. chlamydosporia* was observed to thrive in fields with increased input of organic matter (Viaene and Abawi, 2000). Studies aimed at characterizing how soil amendments alter the soil microbiome coupled with modern *in vitro* culture and molecular analyses of affected nematophagous fungi should enable detailed studies to dissect the major factors contributing to these indirect effects.

## Conclusions

Soil is a very complex environment with many interacting partners; these interactions are hence multifaceted and complex. A good biocontrol agent against SCN should not only be able to parasitize and/or produce toxins *in vitro*, but must also do the same in soil, while overcoming the effects of other microbes present in the soil. Several fungal genera have been routinely isolated from the SCN and have shown potential antagonism. In the absence of effective chemical control methods, screening and selecting good biocontrol agents that can thrive in several soils and effect biocontrol will complement the cultural practices already in place. It is noteworthy that a majority of culturable fungi for the SCN belong to the order Hypocreales and the family Nectriaceae, and their presence in all SCN soybean fields studied indicates possible soybean-SCN-fungal tri-partite interactions and co-evolution between these organisms. It is also evident that these fungi use both parasitism and toxicity as mechanisms to tackle SCN. Apart from fungal organisms directly employed for biocontrol, specific compounds produced by these organisms could become future biopesticides. In this multifaceted scheme of interactions, a more thorough understanding of the effects of organic amendments to soil on microbial communities will aid in effective management of these potential biocontrol organisms. Known fungal taxa associated with the SCN are a starting point for future investigations to build on existing knowledge of biocontrol agents and potential bionematicides to develop more effective strategies for control of the SCN.
